# ESCP best practice: development of the national methodology for the provision of clinical-pharmaceutical care and its implementation in the healthcare system of the Czech Republic

**DOI:** 10.1007/s11096-025-02009-8

**Published:** 2025-09-30

**Authors:** Jan Miroslav Hartinger, Ivana Tašková, Jana Gregorová, Ondřej Slanař, Daniela Fialová

**Affiliations:** 1 Institute of Pharmacology, 1st Faculty of Medicine, Charles University and General University Hospital, Prague, Czech Republic; 2 Department of Clinical Pharmacy, Psychiatric Hospital Bohnice, Prague, Czech Republic; 3 Department of Clinical Pharmacy, Faculty Hospital Bulovka in Prague, Charles University, Prague, Czech Republic; 4 Department of Social and Clinical Pharmacy, Faculty of Pharmacy in Hradec Králové, Charles University, Hradec Králové, Czech Republic; 5 Department of Internal Medicine and Geriatrics, 1st Faculty of Medicine, Charles University, Prague, Czech Republic

**Keywords:** Cost–benefit analysis, Clinical pharmacy, Inpatients, Legislation, Medication review, Medication therapy management, Outpatients, Reimbursement

## Abstract

**Introduction:**

In 2011 clinical pharmacy (CP) almost did not feature in the Czech Republic. As the complexity of pharmacotherapy increased, the need for comprehensive medication reviews (CMR) became increasingly important which led to extension of pharmacy practice beyond merely drug-oriented pharmacy-based services.

**Aim:**

To outline the development, implementation and outcomes of the methodology that established standards for CP practice in the Czech Republic and which contributed to establishing CP as an independent postgraduate specialization with its own workplaces and full-time employment positions.

**Setting:**

Inpatient and outpatient healthcare settings in the Czech Republic.

**Development:**

Legislative changes in 2011 incorporated CP care into the healthcare system and the national CP methodology was published in 2014. Proactive screening of the medication lists and patient healthcare documentation was introduced. Results of CMRs are discussed with attending physicians and the plans for drug therapy adjustment are added to patient documentation. Clinical pharmacists have become standard partners for physicians on medical wards and outpatient facilities. A comprehensive clinical postgraduate training program (fully interlinked with accredited CP wards) has been established to maintain high standards of CP care.

**Implementation:**

Based on the CP care methodology approved by professional medical and pharmaceutical societies and accepted by the Ministry of Health and health insurance companies, three inpatient procedures and one outpatient procedure became eligible for reimbursement thus facilitating the further development of CP practice and independent CP departments. Currently, the Czech Republic has 58 CP facilities and nearly 200 specialized clinical pharmacists.

**Evaluation:**

The provision of CP care according to current national guidelines was shown to provide an effective and cost-effective approach by the results of two extensive studies; the calculated economic cost–benefit ratio was determined at 1:3–4.2. The number of clinical pharmacy specialists and facilities is steadily increasing.

**Conclusion:**

The development of methodological approach accompanied by changes concerning reimbursement in the Czech Republic have led to the establishment of a stable and well-defined environment for clinical pharmacists to become full-time experts in both inpatient and outpatient settings. Clinical pharmacists are now recognized as skilled experts who are respected for their valuable contribution to inter-professional cooperation within medical teams.

## Facilitators of best practice

- The legal introduction of “clinical-pharmaceutical care” and other related legislative, healthcare and reimbursement changes, including the reimbursement coverage of the procedures provided by specialists in clinical pharmacy firstly in the acute inpatient care setting and, subsequently, in the outpatient setting.
- The establishment of mainly pharmacy-independent clinical pharmacy departments that focus exclusively on the provision of clinical pharmacy services in both the inpatient and outpatient setting.
- The 5-year full-time training of clinical pharmacists at accredited clinical pharmacy departments in various regions of the Czech Republic.

## Barriers to best practice

- The misconception that services provided by pharmacies largely overlap with those provided by clinical pharmacy departments even though the purpose of these services is different, and skill requirements for the provision of CP services differ significantly from those needed in pharmacies. This barrier has largely been overcome in the Czech Republic via the 3 facilitators listed above and via the acceptance that these two services differ substantially.
- The uncertainty felt by other pharmacy professionals, particularly “community pharmacists” and “hospital pharmacists” that the independent development of clinical pharmacy will undermine their professional expertise. Paradoxically, the opposite is true since specialists in clinical pharmacy often provide training for community and hospital pharmacists and help them to enhance their expertise.
- CP should be introduced into more legislative standards so as to ensure its continued integration into the healthcare system. This factor depends on support from the Ministry of Health and its realization will only come about via discussions with the decision-making bodies representing other pharmacy specializations and wider medical professional communities.

## Background

The prevalence of multimorbidity and polypharmacy in the population are increasing globally as are the complexity of pharmacotherapy and the risks of drug-related problems (DRPs) [1]. As a consequence, the routine medication safety checks conducted by dispensing pharmacists or pharmacists consulting in pharmacies armed with limited clinical skills and information no longer fulfill the requirements of the comprehensive medication reviews (CMR) of the complex therapeutic regimens [2]. The main aim of clinical pharmacy (CP) is to optimize the utilization of medicines through practice and research in order to achieve set person-centered and/or public health objectives. CP should ensure the attainment of health-related person-centered objectives with a focus on optimizing the utilization of medicines so as to guarantee maximal effectiveness and safety and the term CP also encompasses a raft of skills and services provided by pharmacists that extends beyond their traditional role (the development, production and dispensing of drugs) and shifts the focus from the drug to the needs of patients [3]. Thus, clinical pharmacists are not involved in logistics, storage and other pharmacy-based issues, but are fully committed to the pharmacotherapeutic needs of individual patients. Consequently, clinical pharmacists comprise valuable healthcare team members, the responsibilities of whom include addressing issues surrounding increasing levels of polypharmacy and interactions between drugs, and ensuring e.g. the appropriate, highly individualized dosing and the appropriate medication combinations for patients treated by multiple specialists [4]. Nevertheless, in order to ensure high quality clinical-pharmaceutical care, the scope of the knowledge and skills of pharmacists must be dramatically reshaped, including the requirement for a thorough postgraduate education and, ideally, extensive pre-graduate clinical training.

Even though CP already comprises a well-established healthcare specialization in many countries, it remains either under-evaluated or even practically non-existent in many European countries [4]. This was the case in the Czech Republic in 2011. However, within just over a decade, due to major legislative, educational and clinical developments, not only major acute care hospitals but also many outpatient facilities in the Czech Republic now employ full-time clinical pharmacists. The introduction of full-time stable positions for, and the rigorous education of, clinical pharmacists has allowed both for the delivery of patient-centered clinical-pharmaceutical care services and the conducting of multiple research projects in the field of CP and clinical pharmacology [5–17]. Nevertheless, the development of CP as a scientific discipline that arose from the needs of daily clinical practice is only marginally noted in this article. Rather, this article focuses primarily on the various achievements in the development of clinical pharmacy practice in inpatient and outpatient medical facilities in the Czech Republic aimed, *inter alia*, at inspiring other EU and non-EU countries.

### Aim

The aim of this article is to provide a summary of the significant development of CP in the Czech Republic in the period 2011–2024, commencing with an outline of the legislative changes that enabled the establishment of pioneering CP wards that allowed for the development of a comprehensive clinical CP methodology, and continuing with a description of the implementation of CP services in acute care hospitals and outpatient facilities in the Czech Republic.

### Development and implementation of clinical-pharmaceutical care in the Czech healthcare system

#### Legislation

**Fig. 1:**
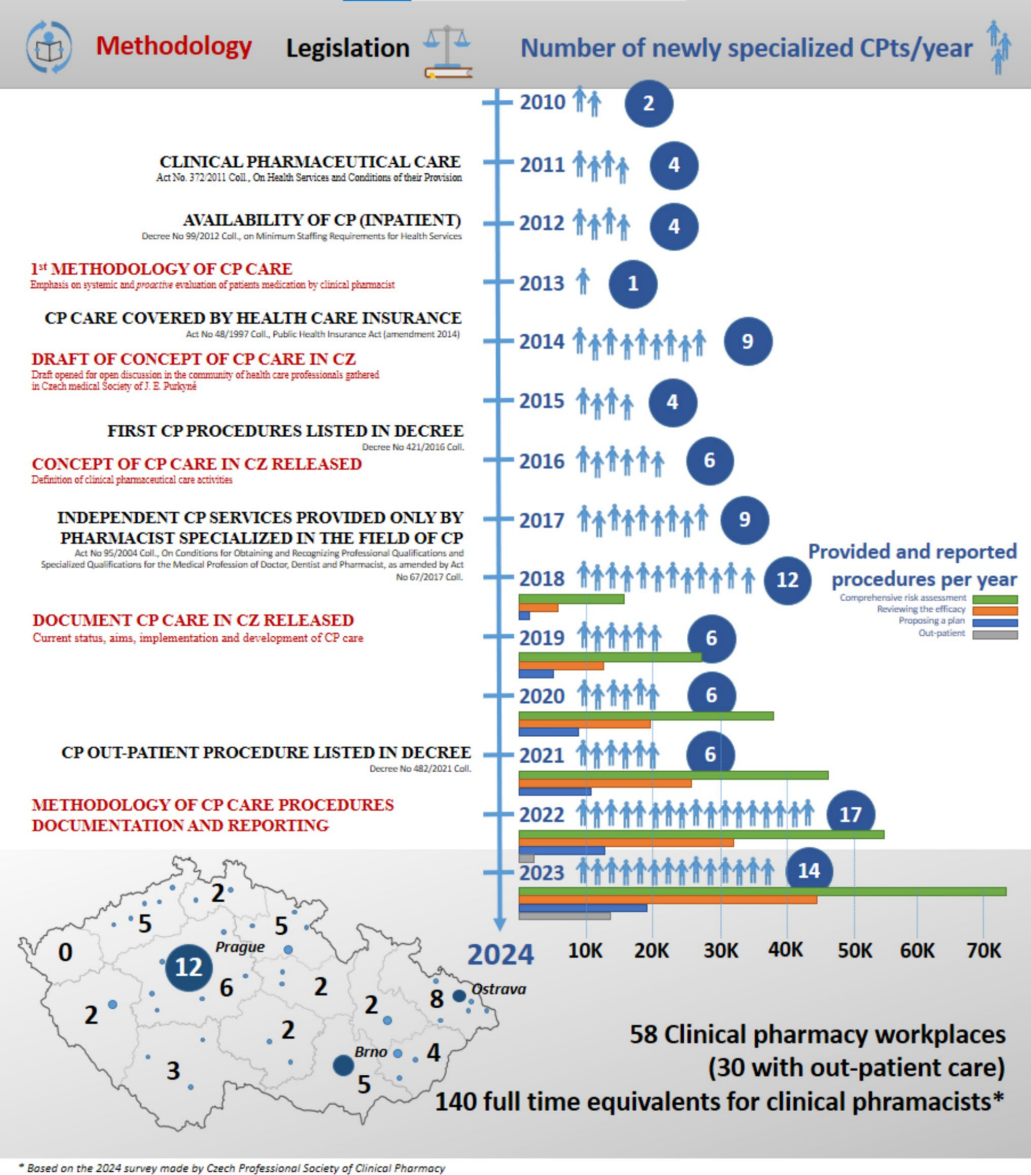
Development of clinical-pharmaceutical care services in the Czech Republic from 2010 to 2024

The important legislation that paved the way for the availability of CP services for patients in the Czech Republic is listed in the left part of Fig. 1 together with the related methodological documents published by the Czech Professional Society of Clinical Pharmacy. The respective Czech-specific legal references and key legislation are listed with explanations in Table 1. The first major legislative development in terms of the establishment of CP as a clinical field comprised Act No. 372/2011 Coll. (2011) that distinguished patient oriented *clinical-pharmaceutical care* (in Czech* “klinicko-farmaceutická péče”*), which corresponds to the term “pharmaceutical care” in the European Society of Clinical Pharmacy (ESCP) CP definition [3], as a fully patient-oriented CP service that can be provided outside pharmacies. Pharmacy-based services involving logistics, preparation and dispensing of drugs are regarded as pharmacy-based pharmaceutical care (in Czech *“lékárenská péče”*) [18]. This pharmacy-based care service remains primarily more drug- than patient-oriented and is provided by the majority of Czech pharmacies. From 2012 clinical-pharmaceutical care was guaranteed for hospitalized patients via Decree No. 99/2012 Coll.

**Table 1:** Brief explanation of key Czech-specific legal references

Name of the legislation	Short explanation of the content	Importance for clinical-pharmaceutical care
*Act No. 372/2011 Coll* Act on Health Services and Conditions of their Provision (Health Services Act)	This Act regulates health services and the conditions of their provision and the related exercise of state administration, types and forms of healthcare, and the rights and obligations of patients and persons close to patients, health service providers, health professionals, other professional staff and other persons in connection with the provision of health services, the conditions for assessing the quality and safety of health services, other activities related to the provision of health services and incorporates the relevant European Union regulations	Definition of patient-oriented *clinical-pharmaceutical care*
*Decree No. 99/2012 Coll* Decree on Minimum Staffing Requirements for Health Services		The decree sets out the requirement for the availability of clinical pharmacists in inpatient healthcare facilities that provide standard and intensive acute care services
*Act No. 48/1997 Coll* Act on Public Health Insurance and on Amendments and Additions to Certain Related Acts	This Act regulates public health insurance (“health insurance”), the scope and conditions under which health services are covered by health insurance, the method of determining prices and reimbursement of medicines and foodstuffs for special medical purposes covered by health insurance, method of determining reimbursement for medical devices and in vitro diagnostic medical devices prescribed on a voucher covered by health insurance	Insurance coverage of clinical-pharmaceutical care (2014 amendment)
*Decree No. 421/2016 Coll.* which details a list of medical services accompanied by point values (i.e. covered by insurance)		The first three CP procedures for inpatients are listed accompanied by a financial evaluation. The decree further contains a list of risk factors that standardizes the stratification of patients according to the risks associated with their pharmacotherapy
*Decree No. 482/2021 Coll* which details a list of medical services accompanied by point values (i.e. covered by insurance)		Includes the first CP procedure for outpatients
*Act No 95/2004 Coll* On Conditions for Obtaining and Recognizing Professional Qualifications and Specialized Qualifications for the Medical Professions of Doctor, Dentist and Pharmacist, as amended by Act No 67/2017 Coll	This Act regulates the conditions for obtaining the qualification to practice the medical profession of a physician, dentist and pharmacist in the Czech Republic, the lifelong education of a physician, dentist and pharmacist and the recognition of the qualification to practice the medical profession of a physician, dentist and pharmacist	Independent clinical pharmaceutical services can be provided only by pharmacists who specialize in the field of clinical pharmacy*

*This term should not be confused with “hospital pharmacist”, which comprises a completely different specialization in the Czech Republic

Healthcare insurance coverage for clinical-pharmaceutical care was established in 2014 via an amendment to Act No. 48/1997 Coll.; and in 2016 the Decree No. 421/2016 Coll. defined three CP procedures that are financially reimbursed, details of which are provided in Table 2 along with an outpatient procedure that was added via Decree No. 482/2021 Coll. Decree No. 421/2016 further included a list of risk factors that served to standardize the stratification of patients according to the risks associated with their pharmacotherapy (Table 3). The number of procedures allowed for one patient during one hospital stay is limited. Patients at medium and high risk are entitled to additional CP procedures. Payment for these procedures is usually collected by the healthcare provider (i.e. the hospital), which then reimburses clinical pharmacists.

**Table 2:** List of clinical-pharmaceutical procedures covered by health insurance in the Czech Republic in 2024

Procedure	Evaluation	Duration	Note
Comprehensive assessment of the medication risks of patients by a clinical pharmacist	272 CZK (10.9 EUR)	15 min	A type of advanced medication review provided for patients with risk factors as defined in decree 421/2016 Coll. (Table 3). These patients are regarded as medium risk if no action is required (i.e. only the risk factors have been identified) or high risk where action is required and a plan for medication adjustment is drawn up (i.e. with or without risk factors but in all cases with a medication adjustment plan proposal). This procedure is not recorded for low-risk patients without risk factors who do not need a plan for medication adjustment Clinical pharmacists work with patient healthcare documentation and laboratory results, and in case of need discuss problem issues with the attending physician and the patient. This procedure is routinely provided for all medium and high-risk patients on wards that implement the “comprehensive CP care model” and potentially triggers a proposal for a plan for medium and high risk patients
Proposing a plan for adjusting patient medication by a clinical pharmacist	362 CZK (14.5 EUR)	20 min	This procedure is provided when patients are placed in the high-risk group during the *Comprehensive assessment of the patient’s medication risk by a clinical pharmacist*. Regarding medium-risk patients, the plan is proposed only in the case of the expected need for medication adjustment during hospitalization and for patients initially evaluated as low risk only in the case of a change in their health condition A plan may also be proposed without the prior conducting of a *Comprehensive assessment of the patient’s medication risk by a clinical pharmacist* on the direct request of a physician, or according to the criteria defined for the particular ward/facility (e.g. always when a drug with a narrow therapeutic index is prescribed, etc.)
Reviewing the effectiveness of proposed medication changes by a clinical pharmacist	362 CZK (14.5 EUR)	20 min	This procedure is provided only after the proposed plan for medication adjustment has been accepted
Evaluation of the pharmacotherapy of outpatients by a clinical pharmacist	272 CZK (10.9 EUR)	15 min	This corresponds to an outpatient “advanced medication review”. It may be reported up to four times daily to cover a maximum of sixty minutes of work per one patient (depending on the complexity of the medication problem). The maximum number of procedures provided/reported for one patient is eight per year

**Table 3:** Risk factors for stratifying patients based on the risk associated with their pharmacotherapy (as defined in Decree 421/2016 Coll.)

Polypharmacy—more than eight drugs Drugs with a narrow therapeutic window (vancomycin, aminoglycoside antibiotics, phenytoin, carbamazepine, valproic acid, warfarin, low molecular weight heparin (LMWH) in therapeutic doses, cyclosporine, everolimus, tacrolimus, temsirolimus, digoxin, theophylline and possibly other drugs whose plasma levels need to be monitored when adjusting the dosage, in case of the alteration of eliminating organ function, when an adverse effect occurs or when monitoring the impact of drug interaction) Drugs with high interaction potential Renal impairment—eGFR less than 30 mL/min Liver impairment—albumin less than 20 g/L, increased ALT, AST, GMT, bilirubin Significant changes in other biochemical and/or hematological parameters Patients in ICU Diabetes mellitus on pharmacotherapy (either insulin or non-insulin antidiabetics) Epilepsy treated with anticonvulsants Atrial fibrillation Cancer—curative or palliative pharmacotherapy Treatment with corticosteroids/other immunosuppressant > than 1 week Patients with Parkinson’s disease	

eGFR, estimated glomerular filtration rate; ALT, alanine transaminase; AST, aspartate transaminase; GMT, glutamate transferase; ICU, intensive care unit

#### Methodology of clinical-pharmaceutical care in the Czech Republic

The Czech CP care methodology was inspired by CP practice abroad, particularly by the approach of American College of Clinical Pharmacy and its definition of a clinical pharmacist as a specialist who “works directly with physicians, other health professionals and patients to ensure that the medications prescribed for patients contribute to the best possible health outcomes” [19] with an emphasis on the proactive intervention of clinical pharmacists as members of multidisciplinary healthcare teams [20]. The proposed CP care methodology was introduced in Czech Republic only following a thorough evaluation of its effectiveness by a pilot study conducted in 2014 [19]. Subsequent project that further tested proposed methodology was conducted in 2015. This project involved the analysis of the performance of four clinical pharmacists that controlled 3,946 patients and provided a total of 884 drug therapy recommendations encompassing 1595 drug interventions. This served to highlight the ability of clinical pharmacists to reduce medication risks for patients [21].

Also the ESCP commentary on the definition of CP states that clinical pharmacists should proactively search for medication problems and resolve them whenever possible [3], an approach that is followed in the Czech Republic. The preferred CP care model comprises the *“comprehensive CP care model”* according to which clinical pharmacists screen all patients during admission, provide them with medication reviews and subsequently work closely with attending physicians on proposed medication changes. This allows for the evaluation and elimination of potential medication risks prior to the occurrence of drug-related complications. The clinical pharmacist estimates the patient’s medication risk, recommends changes to the patient’s medication therapy and proposes a plan for further controls. During the controls the clinical pharmacist verify the effectiveness of the changes. This approach reflects the fact that only clinical pharmacists are able to accurately estimate and assess the amount of CP care required by patients. Nevertheless, due to the still relatively small number of clinical pharmacists, it is not yet possible to implement the *comprehensive CP care model* in all hospitals; hence the introduction of the *“selective CP care model*”. According to this model, patients admitted to hospital are selected for a medication review according to predefined risk factors (e.g. CP care is provided only for patients with kidney impairment or those on interacting drug medication). The third and least preferred model applied in the interim period before enough clinical pharmacists are available comprises consultation on request only.

The methodological documents cited in the following paragraph were compiled and published by the Czech Professional Society of Clinical Pharmacy and were closely linked to legislative changes (see Fig. 1) and influenced by subsequent amendments to the relevant legislation. The draft of the “Concept of Clinical-Pharmaceutical Care in the Czech Republic” was released in 2014 [22], i.e. the same year as the introduction of an amendment to Act. No. 48/1997 Coll., which introduced the health insurance coverage of CP care. The draft was scrutinized by medical and other pharmaceutical specialization experts and comments were submitted from a wide spectrum of professional societies. Subsequently, the first official version of the CP care concept, which addressed the first three CP inpatient procedures listed in Table 2, was published in 2016 [23]. The concept was then adapted so as to fully take into account the content of European Statements of Hospital Pharmacy [24] and the development of CP in other countries (particularly the USA and the UK) and newly published in 2019 [25]. It should be noted that CP and Hospital Pharmacy comprise two distinct specializations in the Czech Republic and hospital pharmacists do not have the education and competencies required to provide the full range of CP care services.

#### Education of specialists in clinical pharmacy

Several studies have shown that simple and short education courses provided for community and hospital pharmacists [26] or strategies involving the use of simple patient education leaflets [27] are insufficient in terms of ensuring highly individualized pharmacotherapy and improvement of patient  outcomes. Indeed, comprehensive training and education in all the areas involved in the daily practice of clinical pharmacists is the only way to ensure the responsible provision of effective clinical-pharmaceutical care. The subjects related to CP are already taught as part of undergraduate courses at both Czech pharmaceutical faculties [18], however, only the postgraduate CP specialization program teaches pharmacists the basic clinical skills essential for independent clinical decision-making and patient interventions.

After graduation, pharmacists in the Czech Republic has to continue in mandatory 1.5 year practice in pharmacies. Then candidates for the specialty in clinical pharmacy can start a CP postgraduate course. This CP postgraduate course is certified by the Ministry of Health and ensures that all independently practicing clinical pharmacists are armed with the skills that allow them to be fully accountable for the advice and recommendations they provide. As part of the CP postgraduate training program, students work for 3.5 years under the supervision of specialized clinical pharmacists on accredited CP hospital wards (accreditation is granted by the Accreditation Committee of Clinical Pharmacy of the Ministry of Health) and are required to attend the mandatory courses on multiple drug-therapy topics (e. g. rational antibiotic therapy, drug therapy of patients with liver or kidney insufficiency etc.). Subsequently, participants are required to write a short theoretical work on chosen area of their clinical interest, which they are required to defend during the final examination, which comprises also the analysis of a model case report (practical part) and questions on specific medical topics (theoretical part).

Following qualification, clinical pharmacists are permitted to practice independently, i.e. without supervision and to provide the full range of CP services. Following a further two years of clinical practice, they become eligible to supervise the specialization program. The CP postgraduate program is wide-ranging and, despite its challenging nature, the number of participants is increasing steadily, as shown in Fig. 1. The average number of newly-specialized clinical pharmacists per year increased from 4.3 in the period 2010–2016 to 10 in the period 2017–2023.

### Evaluation of the efficacy and cost-effectiveness of the clinical-pharmaceutical care provided by clinical pharmacists in the Czech Republic

The benefits of high-quality comprehensive CP services have been repeatedly demonstrated in other countries and various healthcare systems and settings [28]. It has even been shown that CP services in community settings reduce hospitalization rates [29]. The first study on the evaluation of the cost-effectiveness of CP services in the Czech Republic was conducted in 2014 and demonstrated an economic cost–benefit ratio of 1:3 [19]. Subsequently another study demonstrated the cost–benefit ratio 1:4.2 in the setting of an inpatient psychiatric hospital [30]. The general characteristics of these studies are listed in Table 4.

**Table 4:** Studies that have evaluated the cost-effectiveness of clinical-pharmaceutical services in the Czech Republic

Study duration and setting	Number of subjects	Outcome	References
One year (2014); five departments in a large university hospital (general surgery, infectious diseases, oncology, orthopedics, thoracic surgery, and respiratory medicine); four clinical pharmacists	9153 patients screened; 1916 interventions by clinical pharmacists (21% of screened patients)	Calculated economic cost–benefit ratio 1:3	[19]
Over two years (2020–2021); a large psychiatric hospital; employed 1.2 full-time equivalent clinical pharmacists	2,938 patients screened; 1,049 therapy recommendations by clinical pharmacists (36% of screened patients). Each of the drug therapy recommendations featured an average of approximately two clinical pharmacy interventions, which amounted to 2,035 clinical pharmacy interventions related to specific drugs	Calculated economic cost–benefit ratio 1:4.2	[30]

In general, the efficacy of both the methodology and education/training program in the Czech Republic is further illustrated by the increasing number of newly-qualified clinical pharmacists and the increasing number of CP procedures provided per year (Fig. 1). It is worthy of note that it is likely that Fig. 1 underestimates the amount of provided CP care due to the underreporting of procedures to the Institute of Health Information and Statistics of the Czech Republic; nevertheless, the Figure clearly illustrates the steady increase in the numbers of reported procedures.

### Research in clinical pharmacy in the Czech Republic

#### Practice-oriented research in clinical pharmacy in the Czech Republic

The cooperation with other medical fields has contributed to generating the data and to conduct meaningful research projects. Therefore, clinical pharmacists start to study e.g. the pharmacokinetics of drugs so as to provide the background for the creation of rational dosing schedules [5–7]; develop tools for evaluating [8] and improving patient adherence [9] and work together on publishing inter-professional clinical guidelines [31]. Clinical pharmacists also frequently publish articles in the Czech medical press aimed at the rationality of drug therapy and boosting interdisciplinary cooperation. The average annual number of scientific and professional publications involving clinical pharmacists in the period 2000–2024 amounted to 45, around half of which were published in international journals.

#### Academic research in clinical pharmacy in the Czech Republic

Academic research in the field of CP in the Czech Republic has focused on European multicentric grants in individualized pharmacotherapy and helped to establish cooperation with international policy organizations (e.g. WHO, UNECE, Age Platform Europe) to intensify political support for development of CP nationally and internationally. Particularly international research projects on the individualization of drug schemes in multimorbid older adults has been successful in obtaining funding from the European Commission and are being addressed by Research Unit “Ageing, Polypharmacy and Changes in the Therapeutic Value of Drugs in the Aged” at the Faculty of Pharmacy, Charles University [32], in which Czech CP researchers participated as leading experts or collaborators (see Table 5).

**Table 5:** Czech CP academic research—list of international projects involving participation of Czech CP experts (*)

*Obtained EU grants (2001–2024)* EC FP5 (European Commission Framework Program 5) ADHOC—AgeD in Home Care project (2001–2005)—evaluation of the rationality of drug prescribing in older home care clients in 8 EU countries EC FP7-PREDICT—Participation of the Elderly in Clinical Trials project (2009–2011)—evaluation of the barriers and facilitators for participation of older adults in clinical trials EC FP7-SHELTER—Services and Health in the Elderly in Long-Term Care project (2009–2014)—evaluation of the rationality of drug prescribing in older nursing home residents in 7 EU countries and Israel EU COST Action IS1402 project “Ageism—international, interprofessional perspectives”, Work package 1b (WP1b) “Healthy Clinical Strategies for Healthy Ageing” (2015–2017)—analyses of the availability and prescribing patterns of potentially inappropriate medications in older patients in Central and Eastern Europe. This project established the research collaborative CP network in 6 Central and Eastern EU countries [40, 41] EU Horizon 2020 EuroAgeism H2020 project– ESR7 program on “Inappropriate prescribing and availability of medication safety and medication management services in older patients in Europe and other countries” (2017–2022) [10–14]—participation of 8 EU countries including Czech Republic and 2 developing countries (India and Ethiopia), established collaboration of CP researchers with a number of international policy organizations, including the World Health Organization (WHO), United Nations’ Economic Commission of Europe (UNECE) and Age Platform Europe EU Horizon 2020 I-CARE4OLD project (2021–2025) [15, 16]—the first EU project developing AI models for the prediction of impact of various pharmacological and non-pharmacological interventions on health trajectories in multimorbid older adults, participation of 7 EU countries including Czech Republic, and Canada, USA, Hong Kong and New Zealand *Currently submitted international projects (2025)* Prim-CARE4OLD project- “Primary Care-Based Integrated Community Care for Older Adults”—coordinator: Canada, consorcium including the Czech Republic and 6 EU countries, the EU call “Transformation of HealthCare Systems” IQOLD project- “Improving the quality of life of people living with moderate and severe dementia during their long-term care trajectory: uncovering effective interventions from real-life data”—coordinator: the Netherlands, 6 participating countries including the Czech Republic, EU call “Joint Program on Neurological Disorders” EU Horizon 2020 CHECK4OLD project- “Enhancing the care coordination of older adults with multiple long-term conditions by self-checks in Africa, Canada and Europe”—coordinator: the Netherlands, participation of 10 EU countries including the Czech Republic and 8 African countries,, EC Horizon 2020 call	

(*)Research Unit “Ageing, Polypharmacy and Changes in the Therapeutic Value of Drugs in the AgeD”, Faculty of Pharmacy, Charles University, Czech Republic [32]

Czech academics have also been active over the long-term in the Research Committee and General Committee of the ESCP [33, 34], as well as in the Special Interest Groups of the ESCP. In 2023, the Faculty of Pharmacy, Charles University, submitted, in cooperation with other national research institutions, a large national expert grant entitled Netpharm—New Technologies in Pharmaceutical Sciences. Work package 4 of this grant is devoted exclusively to the development of the first national CP research network involving several large acute and outpatient CP departments/facilities in the Czech Republic [17].

### Future directions of clinical-pharmaceutical care in the Czech Republic

The main challenge in the next years will be to train as many clinical pharmacists as possible aimed at reducing the number of hospital wards that are not not provided with comprehensive CP care. The Concept of CP care published in 2016 defines the personnel requirements for comprehensive CP care as 1 full-time clinical pharmacist for either 50 acute care beds, up to 300 follow up/long-term beds or up to 200 outpatient physicians. This makes the ideal number of full-time clinical pharmacist positions 1,300 and the ideal number of CP wards 188. Nevertheless, we are still far from these numbers as the current head count is 140 clinical pharmacist full-time equivalents working in 58 CP wards, as referred to in Fig. 1.

The capacity of the education system is limited by the number of active clinical tutors/supervisors for whom the theoretical maximum capacity is currently around 30 new specialists per year. Nevertheless, the capacity of the education system is expected to grow steadily together with the number of newly-specialized clinical pharmacists that will potentially work as tutors. Whereas the number of jobs for clinical pharmacists is currently also limited, this number is increasing with the establishment of CP as a useful and cost-effective clinical practice.

In addition, clinical pharmacists are gradually specializing in particular medical fields according to the wards on which they work (i.e. nephrology, cardiology, intensive care etc.). Although this development has not yet been formalized, the necessity is becoming clear for the establishment of CP specializations in the future. Workshops are already organized on a regular basis on the topics of e.g. therapeutic drug monitoring, extracorporeal eliminating methods, CP in ICU etc.

## Discussion

This article aimed to provide a comprehensive overview of the successful development of the services of clinical pharmacists as specialists in clinical-pharmaceutical care in the Czech Republic in both the inpatient and outpatient care settings. The development of CP services in the Czech Republic has seen the establishment of CP as an independent pharmaceutical specialization that ensures the clinical skills required for the provision of direct patient-oriented care. The introduction of CP to the Czech healthcare system has not always been straightforward and several barriers had to be overcome; nevertheless, a wide range of strategies have been implemented and important milestones attained within a relatively short period of time.

The ESCP definition of CP states that clinical-pharmaceutical care should be provided through “cognitive”, “managerial” and “interpersonal” approaches [3], all of which are ensured by the methodology and overall organization of CP in the Czech Republic:

- A cognitive approach—the application of CP knowledge and skills—is ensured through comprehensive postgraduate training and specialization
- A managerial approach—the development and delivery of services—is reflected in the existence of an independent national professional society and CP wards that are members of the Association of Workplaces and Departments of Clinical Pharmacy established in 2023. The development of CP is further supported by the Accreditation Committee of the Clinical Pharmacy of Ministry of Health, the Department for Postgraduate Training in Clinical Pharmacy of the Institute of Postgraduate Education in Health Care and The University Centre of Clinical Pharmacy
- An interpersonal approach—clinical pharmacy services—is ensured via well-defined procedures classified by health insurance companies as specific services provided only by clinical pharmacists that are covered by the healthcare insurance system

Van Mill et al. in 2001 highlighted particularly a lack of funding, insufficient time and space in pharmacies, the unavailability of clinical data, a shortage of trained staff and the negative attitudes of other pharmacy specializations toward clinical pharmacists as obstacles to pharmacists selecting CP as a pathway of their professional growth. Moreover, inadequate education and skills levels previously prevented pharmacists from taking independent clinical responsibility for patient health and making clinical decisions [35]. We believe that nearly all of the above barriers were, at least partially, overcome during the recent development of CP care in the Czech Republic. In a relatively short time period independent CP wards have been established and clinical pharmacists are now able to focus solely on the full-time provision of CP care outside pharmacies and their work is fully clinically oriented and independent of pharmacy drug-oriented services.

We believe that the establishment of pharmacy-independent CP wards is crucial for the development of CP. Cases exist even today in which private pharmacy chains and, occasionally, even hospital pharmacy managers opine that their employees are “wasting time” on clinical-pharmaceutical care and stress the primary roles of pharmacy staff as devoting maximum attention to rapid drug delivery and logistics services that focus primarily on economic considerations. However, this approach not only limits the time that clinical pharmacists are able to devote to CP services, but also automatically imposes a possible conflicts of interest for clinical pharmacists who work in pharmacies, as their recommendations should be first of all based on current knowledge of medical science and not interconnected with economy issues. Working as clinical pharmacists requires time for clinical training, reimbursement, a high level of proficiency, specialization skills and close inter-professional cooperation with physicians; thus, the profession clearly places different demands to those required for working in pharmacies.

Also other countries that provide CP services independent of the logistic, dispensing and consultation services have witnessed the rapid growth of CP provision. In Slovenia, clinical pharmacists have opened independent CP outpatient clinics in cooperation with GPs, which has paved the way for the establishment of a national program that includes the reimbursement of outpatient CP services [36]. Moreover, clinical pharmacists in the UK followed a similar path via the introduction of independent, fully reimbursed, CP services in the outpatient setting. Such CP services clearly require not only skills in terms of compiling CMRs, but also training in the basic diagnostic skills required of primary care physicians [37]. “Prescribing pharmacists” in the Netherlands work full-time in partnership with GPs and in Canada clinical pharmacists are included in medical teams at specialized outpatient clinics, e.g. heart failure clinics, anticoagulation, pain clinics etc. [38, 39].

In a Dutch setting the clinical pharmacists were trained in out-patient setting and after 15-month training program with GPs they effectively reduced the number of patient hospitalizations [29]. The approach in the Czech Republic is rather the opposite, i.e. clinical pharmacists receive comprehensive training in hospital wards, which may be followed by the application of their knowledge during providing the outpatient procedures. This approach mirrors the professional pathways of specialized physicians and we strongly believe that this comprises a more natural approach since the experience required for practicing in the community healthcare setting should ideally be provided by working in hospital wards with complicated patient cases that require the provision of managed and complex healthcare services. The inpatient setting also allows for the more detailed study of patient histories and disease development, as well as a deep understanding of the clinical situations that may lead to patient acute decompensation and following changes to their medication, and thus provides for a broader overview of patient care.

The drawbacks of the current education system include the fact that independent non-pharmacy based CP departments are not permitted to train newly-graduated pharmacists who wish to choose CP specialization since pharmacists need at least 1.5 years of practice in a pharmacy before enrolling on a CP specialization program. While this factor represents a certain anachronism in the Czech education program, it reflects the current pace of the transition from the time of purely pharmacy-based clinical pharmacists to the present period characterized by proactive independent clinical pharmacists. A further problem lies in the fact that the demands placed on clinical pharmacists are much higher than can be provided by the limited number of specialists available and the situation is not likely to improve significantly in the near future. The education program is complex and although the number of clinical pharmacists is increasing steadily, it is still lower than required. This represents a quasi-tax on the high standard of CP care that has been established in the Czech Republic. Nevertheless, we believe that if CP is to develop and maintain its position as a respected specialization, the quality rather than quantity of the services provided should be the main priority of the education program.

## Conclusion

Today, CP in the Czech Republic is a clearly defined specialization with a standard professional methodology and advanced practical research programs based on daily clinical practice, as well as innovative academic research programs in topics related to clinical pharmacy. This paper provides a description of current best practice, which continues to evolve along with the successful introduction of CP services into everyday patient-oriented care in the Czech Republic in both the inpatient and outpatient settings. We demonstrated the cost-effectiveness of CP services through the results of several studies which is in line with positive outcomes of the provision of CP services described in other healthcare systems [28]. Finally, we believe that this comprehensive overview of the development of clinical-pharmaceutical care in the Czech Republic has the potential to provide inspiration for other European countries in which CP remains underdeveloped.

## Data Availability

No datasets were generated or analysed during the current study.
